# Recent Trends in Sedentary Time: A Systematic Literature Review

**DOI:** 10.3390/healthcare9080969

**Published:** 2021-07-30

**Authors:** Hui Fang, Yuan Jing, Jie Chen, Yanqi Wu, Yuehua Wan

**Affiliations:** 1 Library, Zhejiang University of Technology, Hangzhou 310023, China; fanghui@zjut.edu.cn (H.F.); chjie@zjut.edu.cn (J.C.); wuyanqi@zjut.edu.cn (Y.W.); 2 Institute of Information Resource, Zhejiang University of Technology, Hangzhou 310023, China; 3 Library, Zhejiang Sci-Tech University, Hangzhou 310018, China; jingyuan@zstu.edu.cn

**Keywords:** sedentary time, bibliometric, COVID-19, physical activity, children

## Abstract

This paper systematically reviews and synthesizes the relevant literature on sedentary time research. A bibliometric analysis was conducted to evaluate the publications from 2010 to 2020 in the Web of Science (WoS) core collection database. Derwent Data Analyzer software was used for the cleaning, mining, and visualization of the data. Historical trends of the topics, main contributors, leading countries, leading institutions, leading research areas, and journals were explored. A total of 3020 publications were studied. The United States, the United Kingdom, and Australia are the three most productive countries. The Australian institution Baker Heart and Diabetes Institute led the list of productive institutions, and Ekelund U published the most papers. Sedentary time raised the concerns of scholars from 106 research areas, and public health was the dominant field. Physical activity, accelerometer, children, and obesity were the most frequently used keywords. The findings suggest that sedentary time is rapidly emerging as a global issue that has detrimental effects on public health. The hotspots shifted in the past 10 years, and COVID-19 was the most popular topic of sedentary time research.

## 1. Introduction

Sedentary time is rapidly emerging as a global issue that has detrimental effects on public health [1]. Sedentary behavior is defined as any waking activity with very low (≤1.5 MET) energy expenditure [2]. It is typically characterized by time spent sitting or screening in various domains of life, including leisure [3,4], occupation [5,6], and transportation [7,8].

The first serious discussions and analyses of sedentary time emerged in the 1960s. A study published in 1965 sought to determine the relationship between the sedentary time and blood pressure of railroad employees aged 40 to 49 [9]. The effect of exercise on systolic time intervals in sedentary individuals and rehabilitated patients with heart disease was studied in 1971 [10]. The results of a study of the epidemiology of brain infarction associated with occlusive arterial disease showed that sedentary living may be a cause of atherosclerosis [11]. Over the past decades, scholars worldwide have employed epidemiological research methodologies or combinations of experimental designs [12,13], such as cross-sectional studies [14,15,16], longitudinal studies [17,18,19], cohort studies [20,21,22], intervention studies [23,24,25], and quasi-experimental studies [26,27,28], to develop population-based research. Accelerometer-based measurements or self-reported methods are widely used to assess the attribution of sedentary time and light or moderate-to-vigorous physical activity, and samples are taken across all ages (children, adolescents, adults, and old adults), genders, countries, and socioeconomic subgroups [29,30,31,32,33]. Previous research has identified that sedentary behavior and physical activity are determined by or correlated with individual socioeconomic status, the environment, and related health policies [34,35,36]. The relationship between a sedentary lifestyle; the amount, intensity, and frequency of physical activity and diabetes, cardiovascular disease, metabolic syndrome, obesity, cancer, and all-cause mortality has been deeply researched and analyzed [37,38,39,40]. Excessive uninterrupted sedentary time is strongly associated with an increased risk of many chronic diseases [41,42,43,44,45]. It is well recognized that approaches to prevent such diseases and promote health benefits should seek to both increase regular physical activity and decrease sedentary behaviors [46].

Quite a few meta-analyses and systematic reviews of observational studies have previously examined the relationship between sedentary time and unfavorable health outcomes [47]. Researchers have reviewed studies on the pattern of sedentary behavior among older adults in care facilities [48], the effectiveness of physical activity and sedentary behavior interventions in reducing sedentary time in adults [49,50], the necessity of reducing occupational sedentary time among general practitioners and other occupations [51,52], and the effects of sedentary behavior interventions on biomarkers of cardiometabolic risk and asthma [53,54]. However, there is little information available in the literature that presents a distinct perspective by conducting bibliometric analysis to summarize the overall view of the sedentary time research field. Bibliometric analysis is widely used as a valid tool to quantitatively evaluate the distribution of active countries, institutions, authors, and collaborations, and find the hotspots and the research trends in various research areas such as chemistry [33], mechanical engineering [55,56], energy [57], optics [58], software engineering [59], medicine [60,61,62], health [63], economics [64,65], art [66], management [67], urban planning [68], social work [69], etc.

This paper aims to provide a general overview of the sedentary time research area organized as follows: (1) leading countries, institutions, authors, journals and research areas; (2) collaboration patterns between countries and institutions; and (3) research trends and hotspots. This will be accomplished by analyzing author keywords and highly cited papers.

## 2. Methodology and Data Source

Bibliometric analysis is a statistical evaluation of published papers and academic research. Different from systematic review papers, bibliometric methods can analyze massive papers, and show the overall picture of sedentary research from the perspective of the literature. The analysis was based on publications related to “sedentary time” published during the 2000–2020 period. The data were obtained through the Web of Science (WoS) core collection database in May 2021 using a retrieval query of “sedentary time”, searching the “topic” field, and defining the document type as “article and review”. There are some other related keywords in sedentary time research, but in the pre-investigation, we found that the core literature is within the scope of “sedentary time” retrieval, whereas there are quite a few irrelevant documents in the retrieval results of “sedentary behavior”, “sitting time”, “screen time”, and “sedentary lifestyle”. Finally, 3060 publications were collected from the Science Citation Index-Expanded (SCI-E) and Social Science Citation Index (SSCI). After analysis of the yearly outputs, we found that the number of publications in the first 10 years was extremely small and unstable. Therefore, this study limits the scope of the research to 3020 studies published in 2010–2020. Other related publications may have been excluded as a result of the search restrictions mentioned above. We excluded some publications related to ecology, veterinary sciences, energy fuels, marine freshwater biology in the WOS research field. Papers originating from England, Scotland, Northern Ireland and Wales were categorized under the United Kingdom. The impact factor (IF) for each journal was acquired from the 2019 Journal Citation Reports (JCR). The Derwent Data Analyzer (DDA10.0 build 27,330, Search Technology Inc., Norcross, GA, USA), a statistical analysis tool applied for data cleaning, data mining, and data visualization, was used to process and analyze the data extracted from the 3020 publications and form figures. All publications referring to sedentary time during 2010–2020 were assessed for the following bibliometric indicators: publication outputs, countries, international collaborations, institutions, research areas, journals, authors, most cited papers per year, and author keywords. 

It is worth mentioning that some related papers may not be involved in the results. If there were no required words in the title, abstract, or keywords, some related papers could be ruled out. This issue may produce some deviations and certain limitations.

## 3. Results

**Figure 1:**
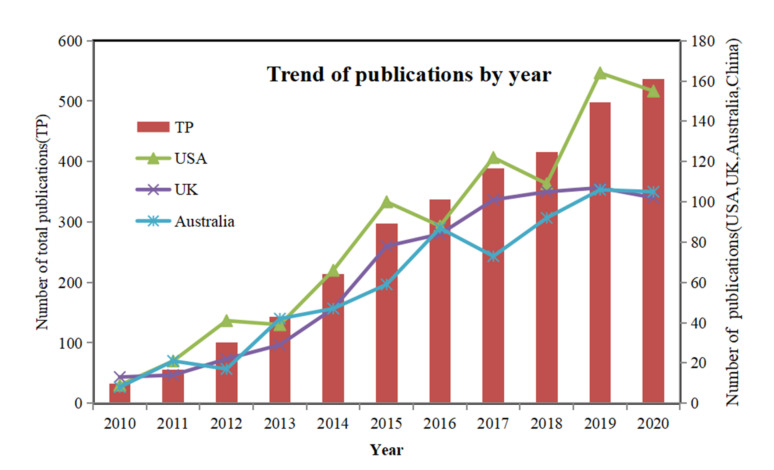
Trends in the number of published articles related to sedentary time by year.

From 2010 to 2020, 3020 publications were contributed by 120 countries/regions to the “sedentary time” research field. Of these 3020 publications, 88 were Essential Science Indicators (ESI) highly cited papers, and 3 were ESI hot papers. The growth trend of sedentary time research over time is presented in Figure 1. The yearly number of publications increased from 32 (2010) to 537 (2020). The total number of studies published on this topic has increased more than 15-fold. The results show that about three-quarters of the literature was published in the last five years (2016–2020), which indicates that sedentary time is an important risk factor impacting public health, resulting in widespread concern among scholars worldwide.

### 3.1. Contribution of Leading Countries/Regions

#### 3.1.1. Number of Publications and Citation

The 3020 publications were from 87 countries/regions. The United States, the United Kingdom, and Australia were the top three most productive countries/regions. The USA was the leader of this field and published 914 sedentary time research papers since 2010. This number was 30% of the total number of publications. The United Kingdom and Australia were also productive in sedentary time research. As shown in Figure 1, the yearly outputs of the three countries/regions grew rapidly between 2011 and 2020.

The number of publications and citations of the top 20 most productive countries/regions in terms of the number of publications related to sedentary time research can be found in Table 1. Of the 20 most productive countries/regions, 13 were from Europe, 3 were from the Americas, 2 were from Asia, and 2 were from Oceania.

The United States, the United Kingdom, and Australia were the three most productive countries/regions, followed by Canada (391), Spain (245), the Netherlands (216), Norway (180), and Belgium (170). Other productive countries included China (153), Sweden (142), Portugal (122), Denmark (121), Brazil (115), Finland (111), and Germany (109). In terms of publishing influence, the United States led the list of total citations (TCs) with 21,980. Despite the high number of publications and citations from the United States, the average citations per publication (ACPP) was relatively low, at only 24.05. Canada led the ACPP rankings at 31.39, and China was in next-to-last place (13.81).

#### 3.1.2. Cooperation of Countries/Regions

Publications were defined as internationally cooperative if the paper was coauthored by researchers from more than one country [70]. As shown in Table 1, of the top 20 countries’ publications, a large proportion were internationally cooperative, especially for Portugal (86.07%) and Belgium (84.71%). This demonstrated that sedentary time raised the concern of scholars worldwide who exchanged ideas with each other. In the 20 countries/regions, 13 European countries comprised a more significant share (over 60%) of papers with international co-authorship relationships. As for SP%, we also observed that the most productive country, the United States, was in next-to-last place (40.37%). The United States was the most active country that had partnerships with 63 countries, followed by the United Kingdom (60) and Spain (47).

**Figure 2:**
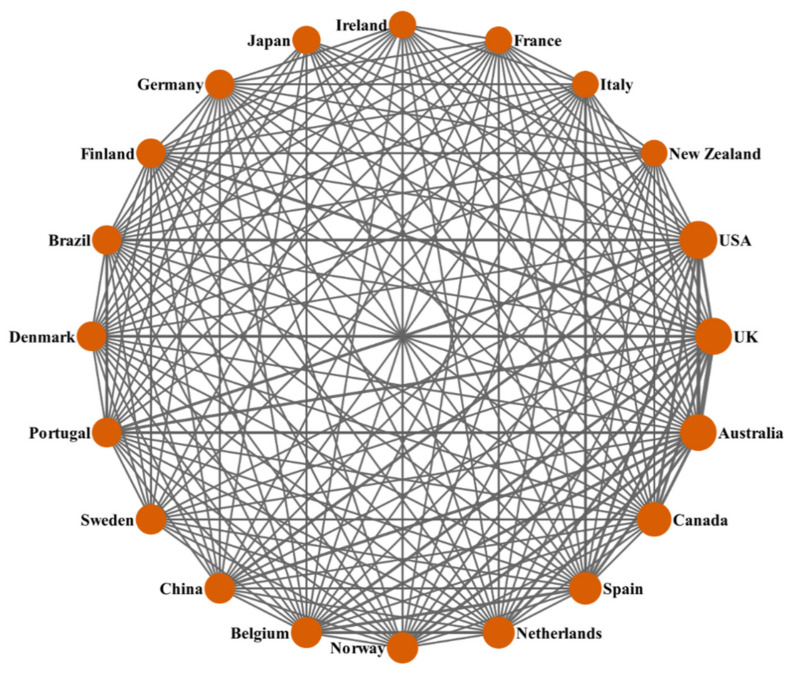
Collaboration matrix map among the top 20 productive countries/regions.

The academic collaboration network of the 20 most productive countries/regions is shown in Figure 2. DDA software was used to draw the network diagram based on the co-occurrence matrix. The size of the circles is proportional to the degree of contribution each country. The lines among these circles represent the cooperation between countries/regions, and the thickness of the lines implies the total number of collaborative publications [71,72,73]. The 20 most productive countries/regions had intensive cooperation with the other countries/regions, especially the United States, the United Kingdom, Australia, and Canada, which were listed in the top four countries/regions.

### 3.2. Contribution of Leading Institutions

The top 20 most productive institutions in sedentary time research, along with their total numbers of publications, citations, and h-indexes, are listed in Table 2. Most of these institutions were from the top three productive countries/regions. Among the top 20 institutions, ten were from Australia; four were from the United Kingdom; and the United States, Belgium, Norway, Spain, the Netherlands, and Sweden each had one. The top three in the list were all Australian research institutions, with the top three h-indexes 47, 45, and 39, respectively. As for ACPP, Monash University located in Australia led the list with 61.39, Loughborough University ranked second with 59.44, and the University of Queensland from Australia was third with 58.58. It was clear that there were no institutions from developing countries. Comparing the research influences of the top 20 institutions that had a prominent role in developing and promoting the field, China, Brazil, and other developing countries still have a long way to go.

**Figure 3:**
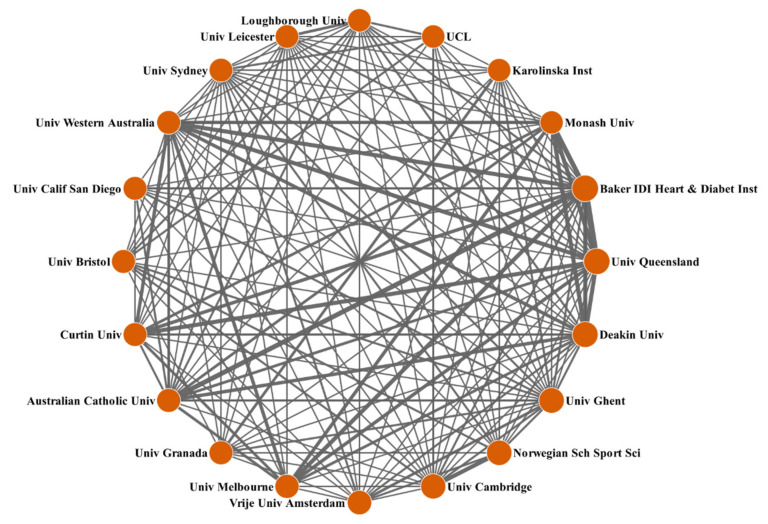
Collaboration matrix map among the top 20 productive institutions.

As shown in Figure 3, institutions from Australia, especially the Baker Heart and Diabetes Institute, the University of Queensland, Deakin University, the University of Melbourne, Australian Catholic University, Curtin University, the University of Western Australia, and Monash University, had a much closer collaborative network among the top 20 most productive institutions. In addition, cooperation among the University of Cambridge, the Norwegian School of Sport Sciences, Ghent University, and Vrije University Amsterdam located in Europe was particularly frequent. This was possibly attributed to the convenience of their geographical locations, promoting the development of collaboration.

### 3.3. Contribution of Leading Research Areas

The research field shown in Table 3 is derived from the core collection of Web of Science, a discipline classification system. Every journal and book covered by Web of Science core collection is assigned to at least one of the subject categories. Every record in Web of Science core collection contains the subject category of its source publication in the Web of Science Categories field. (The WOS research field is detailed in http://images.webofknowledge.com/WOKRS535R102/help/WOS/hp_subject_category_terms_tasca.html, accessed date: 22 July 2021). Sedentary time was a multidisciplinary field covering 106 research areas, and the top 20 research areas ranked by the number of publications are illustrated in Table 3. “Public, Environmental & Occupational Health” dominated the research area listed with 842 papers, followed by “Sport Sciences” (526) and “Nutrition & Dietetics” (359). The results show that the top three research areas comprised nearly 60% of the total publications. The topic was closely related to public health issues; therefore, it was conceivable that most of the top 20 WoS research areas involved various subdisciplines of medicine, and some subjects corresponded to the research of diseases caused by sedentary behaviors, such as “Endocrinology & Metabolism”, contributing a share of 6.92%; “Pediatrics” (5.96%), “Geriatrics & Gerontology” (4.30%), “Cardiac & Cardiovascular Systems” (3.11%), “Oncology” (2.68%), etc. As for ACPP, “Medicine, General & Internal”, “Physiology”, and “Endocrinology & Metabolism” led the list with 43.93, 36.13, and 34.55.

**Figure 4:**
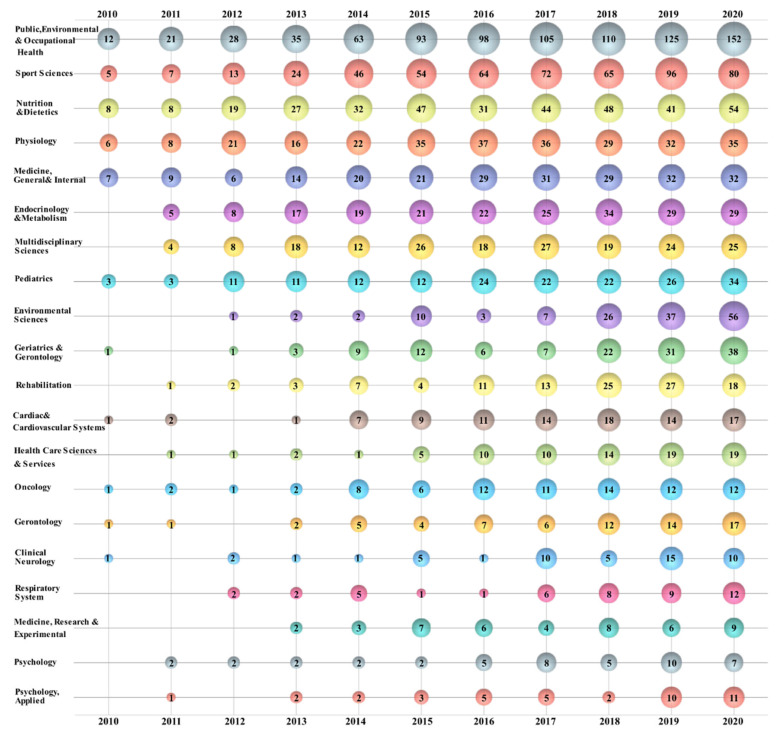
Bubble chart of top 20 sedentary time research areas.

Figure 4 represents a map of sedentary time research. Using visual bubble charts, the development trend of this field is clearly presented in 3D. Note that the number on a bubble represents the number of publications. Researchers from “Public, Environmental & Occupational Health” were the core of sedentary time research. The number of relevant research results increased every year and led the table of yearly publications since 2010. Researchers from “Sport Sciences”, “Nutrition & Dietetics”, “Physiology”, and “Medicine, General & Internal” have long paid attention to topics related to sedentary time, but their output was unstable.

Researchers from “Endocrinology & Metabolism”, “Multidisciplinary Sciences”, “Environmental Sciences”, “Geriatrics & Gerontology”, and other areas became associated with this field later, but the number of published papers grew faster. “Environmental Sciences” entered the table of the top three most productive research areas in 2020. Several research areas, including “Clinical Neurology”, “Respiratory System”, “Psychology”, and others, had sustained output, but their growth was not significant.

### 3.4. Contribution of Leading Journals

Papers related to sedentary time were published in 612 journals. As listed in Table 4, the top 20 journals in terms of the number of publications produced 1385 publications, accounting for 45.86% of the total number of publications during 2010–2020. *BMC Public Health* took the leading position with 170 publications, followed by the *International Journal of Behavioral Nutrition and Physical Activity* (163), *PLoS ONE* (156), *International Journal of Environmental Research and Public Health* (136), *Medicine and Science in Sports and Exercise* (128), and *Journal of Physical Activity Health* (122). The aforementioned six journals comprised 28.97% of the total publications, and the remaining journals had shares of produced papers of less than 2% each. As for total citations, papers from the *International Journal of Behavioral Nutrition and Physical Activity* were cited 7152 times in the past 10 years, followed by *Medicine and Science in Sports and Exercise* (4452) and *PLoS ONE* (4338). Regarding IF, the *British Journal of Sports Medicine* was first with 12.68, and the *International Journal of Behavioral Nutrition and Physical Activity* (6.714) and *Nutrients* (4.546) were second and third, respectively.

It is also worth mentioning that open access publications dominated the study of sedentary time. Among the 612 journals, 581 were open access journals, accounting for 94.93%. Papers published in open access journals amounted to 1977, constituting 65.46% of the total 3020 papers. The top three journals in terms of the number of publications, *BMC Public Health*, the *International Journal of Behavioral Nutrition and Physical Activity*, and *PLoS ONE*, are all OA journals.

### 3.5. Contribution of Leading Authors

Among the 11,249 authors who contributed to sedentary time research, 8195 authors published only one paper, and 9 authors published more than 50 papers. The top 20 most prolific authors based on the number of publications are presented in Table 5.

Ekelund U. led the table with a total of 99 publications; and Owen N. (88), Dunstan D.W. (84), Healy G.N. (64), and Brage S. (61) were ranked second, third, fourth, and fifth, respectively. As for the list of corresponding authors, Cardon G. (20), Hamer M. (15), and Sardinha L.B. (15) were the top three most productive researchers.

For the ACPP, Healy G.N. topped the list with 97.56, followed by Owen N. (87.48) and Dunstan D.W. (80.74). Owen N. achieved the highest h-index of 43, followed by Dunstan D.W. (38) and Healy G.N. (36). Given that the h-index and ACPP, as a reflection of a publication’s quality, can reveal the influence of an author in sedentary time research, it can be seen that Owen N., Dunstan D.W., and Healy G.N., with higher h-indexes and ACPPs, had more significant influences in the field [74].

### 3.6. Research Hotspots and Trends

#### 3.6.1. An Analysis of Author Keywords

The author keywords provide critical information on the current research status and hotspots, and such keywords have been proven to play a key role in analyzing future development trends [75,76,77]. Overall, 3056 author keywords from 3020 papers were analyzed. It is crucial to mention that some publications that did not have author keywords may be excluded from statistical analysis. Among the author keywords, 2027 (66.33%) of the author keywords appeared only once, 429 (14.04%) were used twice and 165 (5.40%) were used three times.

**Figure 5:**
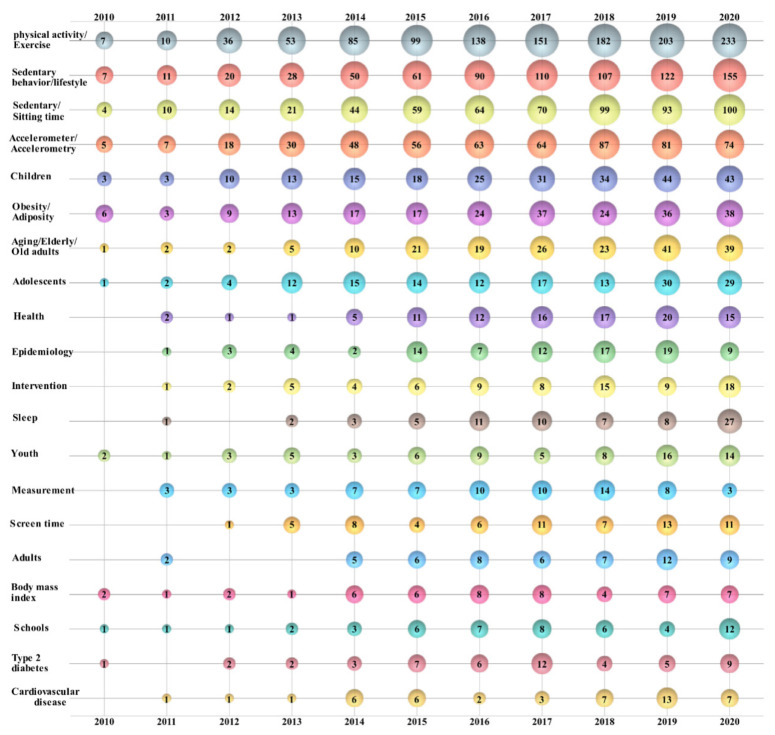
Bubble chart of the top 20 author keywords by year.

The top 20 author keywords by year are shown in a bubble chart (Figure 5). Author keywords with similar meanings were classified as the same item after being cleaned up by DDA.

Except for the search keyword “sedentary time” and the derived keywords “sedentary behavior/lifestyle” and “sedentary/sitting time”, “physical activity/exercise” (1197, first) was the most frequently used term and showed rapid growth since 2012. It was followed by “accelerometer/accelerometry” (533, second), “children” (239, third), “obesity/adiposity” (224, fourth), “aging/elderly/old adults” (189, fifth), “adolescents” (149, sixth), “health” (100, seventh), and “epidemiology” (88, eighth).

#### 3.6.2. An Analysis of Keyword Categories

**Figure 6:**
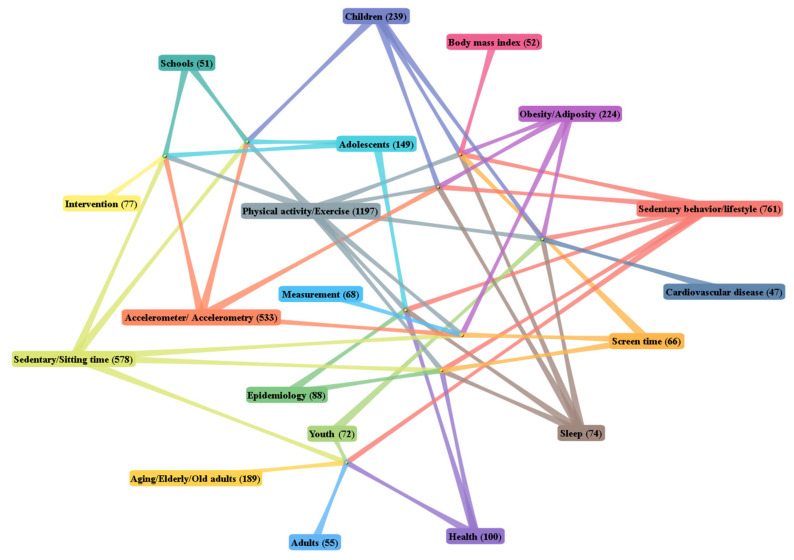
DDA cluster map of the top 20 author keywords.

The top 20 author keywords were chosen to show the relationship between topics (Figure 6). Each node represented a keyword. The data next to the keywords represented the total number of publications from this topic. The lines between the keywords show the co-occurrence of keywords.

As observed from the chart, we divided the top 20 author keywords into the following categories:

1. The measurements used to monitor sedentary time and the effective way to change the pattern of sedentary behavior: physical activity/exercise, intervention, and accelerometry. Accelerometers are commonly used as a device to assess sedentary time; engaging in regular physical activity is widely regarded as a valid measurement to prevent a range of health risk factors across all age, gender, ethnic and socioeconomic subgroups [41,78,79,80,81]. Some intervention studies aiming to increase physical activity or reduce sedentary time have also been conducted [82,83,84];
2. Populations across a wide age range: scholars mainly take samples of children [85,86,87], adolescents [1,81,88], youth [89,90,91], adults [92,93,94], and aging/elderly/older adults [95,96,97], or use different age groups to research sedentary time and patterns of sedentary behavior [98,99,100];
3. Related diseases: the majority of epidemiological evidence has adversely associated high levels of sedentary time and unhealthy sedentary lifestyle with an increased risk of chronic diseases, as listed in the keywords, including obesity/adiposity [92,101], type 2 diabetes [102,103], and cardiovascular disease [104];
4. Biomedical health indicators: body mass index (BMI) is used extensively to select sample ranges, limit sample conditions, and detect changes in sample populations before and after experimental studies [105,106].

#### 3.6.3. An Analysis of the Most Cited Papers

Although the citation impacts of publications will be affected by means of variations [107], it is still a widely used measurement to evaluate scientific publications. The most cited publications in the sedentary time field by year during 2010–2020 are presented in Table 6. The most highly cited publication was “Too Much Sitting: The Population Health Science of Sedentary Behavior” [108] published in *Exercise and Sport Sciences Reviews* in 2010. It was authored by Owen N. et al., and it led the list of total citations with 1214. In terms of TCY, “Sedentary Behavior Research Network (SBRN)—Terminology Consensus Project process and outcome” authored by Tremblay M.S. et al. [109] from Canada ranked first with 203.

Of the most cited publications, five papers had coauthors from institutions in more than one country. Four papers were from institutions in Canada and the United States. Researchers from the United Kingdom contributed to two papers. Institutions from Australia, Norway, New Zealand, Belgium, Brazil, Sweden, and the Netherlands also published highly cited papers. It is worth mentioning that the most cited papers for each year were published in top journals in the field of medicine and sports science, such as the *International Journal of Behavioral Nutrition and Physical Activity* [46], *Diabetologia* and *Medicine and Science in Sports and Exercise* [110]. 

In 2010, Owen N. et al. published a paper showing that sitting time, TV time, and time sitting in automobiles increase premature mortality risk [108]. Following this direction, the relationship between sedentary behavior and health indicators was the hotspot of sedentary time studies during the following years; researchers studied sedentary behaviors and subsequent health outcomes in children, adolescents, and elderly individuals. Researchers have studied the relationship between sedentary behavior and health indicators in children and youth and found that decreasing any type of sedentary time is associated with a lower health risk [111]. Researchers have also examined the association of sedentary time with diabetes, cardiovascular disease and cardiovascular and all-cause mortality; investigated the influence of sedentary behavior on cardiometabolic disease and discovered that regular activity breaks are more effective at decreasing postprandial glycemia and insulinemia [112]; compared physical activity and sedentary time; and studied the effects of sex, age, education, and body mass index [110].

In 2015, Biswas A. et al. published their paper which quantified the association between sedentary time and hospitalizations, all-cause mortality, cardiovascular disease, diabetes, and cancer [47]. Since then, researchers have begun to pay attention to the quantitative analysis of sedentary time and have promoted research in this field. Warburton D.E.R. et al. mentioned that sedentary time is associated with independent health risks and that physical activity should be part of an integrated approach to enhance healthy lifestyle behaviors [43]. Tremblay M.S. et al. raised the importance of standardizing the Sedentary Behavior Research Network (SBRN) due to the need for clear, common and accepted terminology and definitions [109]. The dose–response relationship between sedentary behavior and all-cause behavior was a hotspot of sedentary time research during 2018–2019. Patterson R., Ekelund U. et al. estimated the strength and shape of the dose–response relationship between sedentary behavior and the risk of all-cause, cardiovascular and cancer mortality and incident type 2 diabetes [113], and measured the relationship between physical activity, sedentary time and all-cause mortality [114].

COVID-19 has impacted most people worldwide since 2020. Millions of individuals have been infected with the disease, and billions of individuals have been asked to stay home. Under this background, researchers began to consider COVID-19 as the greatest challenge in sedentary time research. Research by Huckins J.F. et al. analyzed the mental health and sedentary behavior of college students during the early phases of the COVID-19 pandemic, and have been cited 38 times [115].

## 4. Discussion

Eighty-seven countries/regions contributed 3020 papers to sedentary time research from 2010 to 2020, indicating that sedentary time is a global public health issue and attracting worldwide attention. In the past 10 years, the number of sedentary time research papers has increased by 15-fold. A group of researchers and journals focus on this field. The rapid increase in the number publications also revealed the necessity and urgency of sedentary time research.

Currently, increasingly more countries/regions have put efforts into the study of sedentary time. Western Europe, North America, and Oceania were the most active regions in terms of the number of publications. The top 20 countries in the list were all developed countries, except for China and Brazil. The possible reason is that developed countries with high levels of income pay more attention to public and individual health. This was further confirmed by the most active institutions and authors. The majority of the top 20 most productive institutions were from the United States, the United Kingdom, and Australia. Among the 11,249 authors who contributed to sedentary time research, none of the top 20 most productive authors were from China, Brazil, India, or other Asian, South American, and African countries.

The obvious change in the number on the bubble of the author keywords showed the trend of sedentary time research. Physical activity, accelerometers, children, and adiposity were core directions of sedentary time research which have concerned researchers for a long time. In recent years, the number of publications has maintained steady growth. The study of adolescents and old adults started later than that of children, and such papers began to appear after 2013. Currently, sedentary time research has formed the trend of synchronous development of research on children, adolescents, adults, and older adults. Additionally, related diseases such as type 2 diabetes and cardiovascular disease have also been the focus of sedentary time research in recent years.

An analysis of the highly cited papers by year can also conclude that the hotspots of sedentary time research have shifted many times in the past 10 years. Along with the shift of hotspots, significant progress has been made with the work of researchers. Quantitative analysis of sedentary time, including the standardization of sedentary behavior, has become the focus of research since 2015. Since 2020, billions of individuals have been asked to stay at home due to COVID-19, and COVID-19 has become a hotspot of sedentary time research. Stay-at-home orders bring sedentary lifestyles; the relationship among the physical activity, diet, and sedentary time of students, workers, and other groups was studied [116,117].

It is worth noting that sedentary time research involves a wide range of topics including TV time, sitting time, screen time, etc., which cross over with those of other research fields [118,119]. However, there are many differences between them. Sedentary time research is mainly from the public health perspective. Other research fields, such as exploring TV time, are based on theories and methods in market management [120], sociology [121], communication [122], and other disciplines.

## 5. Conclusions

In this study, we analyzed the sedentary time research literature published from 2010 to 2020 based on bibliometrics and the DDA software. The United States, the United Kingdom, and Australia were the three most productive countries/regions. Three Australian institutions, Baker Heart and Diabetes Institute, the University of Queensland, and Deakin University, lead in the table of the most productive institutions. Regarding the subject field, sedentary time research has distinct multidisciplinary characteristics, especially for public health. The top 10 journals in terms of the number of publications, such as *BMC Public Health*, the *International Journal of Behavioral Nutrition and Physical Activity*, and *PLoS ONE*, published 16.19% of the total sedentary time research papers. Ten researchers, represented by Ekelund U., Owen N., and David W.D., published more than 50 papers during the past 10 years. 

Physical activity, accelerometer, children, and adiposity were the most frequently used words. In the past 10 years, sedentary time research has been conducted at various levels, with a series of discussions on the sedentary time of children, adolescents, adults, and elderly individuals. In recent years, researchers have sought to quantify the association between sedentary time and health indicators. Currently, COVID-19 is the most popular topic of sedentary time research.

This study can help potential scholars to better understand sedentary time research on a global scale, providing useful information for relevant scholars to further develop research in this field.

## Figures and Tables

**Table 1:** The top 20 most productive countries/regions in the sedentary time field.

Rank	Country/Region	TP	TC	ACPP	SP (%)	nCC	H-Index
1	USA	914	21,980	24.05	40.37	63	63
2	UK	702	19,649	27.99	66.67	60	64
3	Australia	657	19,413	29.55	71.23	44	66
4	Canada	391	12,274	31.39	49.87	36	51
5	Spain	245	3858	15.75	72.65	47	32
6	Netherlands	216	5949	27.54	65.28	36	38
7	Norway	180	5279	29.33	73.89	40	37
8	Belgium	170	4609	27.11	84.71	42	35
9	China	153	2113	13.81	60.13	37	25
10	Sweden	142	4125	29.05	66.90	31	32
11	Portugal	122	2530	20.74	86.07	41	28
12	Denmark	121	2355	19.46	81.82	38	25
13	Brazil	115	2135	18.57	78.26	42	21
14	Finland	111	1827	16.46	71.17	40	23
15	Germany	109	1607	14.74	68.81	41	20
16	Japan	84	763	9.08	35.71	22	15
17	Ireland	69	1247	18.07	78.26	27	18
18	France	64	1415	22.11	81.25	30	19
19	Italy	58	1164	20.07	81.03	33	18
20	New Zealand	56	1069	19.09	78.57	23	18

TP, total papers; TC, total citations; ACPP, average citations per publication; SP, share of publications; nCC, number of cooperative countries.

**Table 2:** The top 20 most productive institutions in the sedentary time field during 2010–2020.

Rank	Institution	TP	TC	ACPP	h-Index	Country/Region
1	Baker IDI Heart and Diabetes Inst.	165	9328	56.53	47	Australia
2	Univ. Queensland	154	9021	58.58	45	Australia
3	Deakin Univ.	138	5494	39.81	39	Australia
4	Univ. Ghent	125	3970	31.76	34	Belgium
5	Norwegian Sch. Sport Science	122	4279	35.07	31	Norway
6	Univ. Cambridge	116	2883	24.85	28	UK
7	Vrije Univ. Amsterdam	97	3365	34.69	30	Netherlands
8	Univ. Melbourne	88	2695	30.63	29	Australia
9	Univ. Granada	85	1695	19.94	20	Spain
10	Australian Catholic Univ.	84	1603	19.08	22	Australia
11	Curtin Univ.	82	1922	23.44	21	Australia
12	Univ. Bristol	82	2455	30.31	23	Australia
13	Univ. Calif San Diego	81	1505	18.58	22	USA
14	Univ. Western Australia	81	2951	36.43	31	Australia
15	Univ. Sydney	80	2164	27.05	26	Australia
16	Univ. Leicester	76	2884	37.95	22	UK
17	Loughborough Univ.	74	4339	59.44	26	UK
18	UCL	73	2182	29.89	27	UK
19	Karolinska Inst.	71	2220	31.27	24	Sweden
20	Monash Univ.	71	4359	61.39	35	Australia

TP, total papers; TC, total citations; ACPP, average citations per publication.

**Table 3:** Contribution of the top 20 research areas in the sedentary time field.

Rank	WOS Research Area	TP	TPR%	TC	ACPP
1	Public, Environmental & Occupational Health	842	27.88	16,729	19.87
2	Sport Sciences	526	17.42	12,139	23.08
3	Nutrition and Dietetics	359	11.89	10,876	30.30
4	Physiology	277	9.17	10,008	36.13
5	Medicine, General & Internal	230	7.62	10,060	43.93
6	Endocrinology and Metabolism	209	6.92	7187	34.55
7	Multidisciplinary Sciences	181	5.99	4598	25.40
8	Pediatrics	180	5.96	2303	12.79
9	Environmental Sciences	144	4.77	867	6.02
10	Geriatrics and Gerontology	130	4.30	1643	12.64
11	Rehabilitation	111	3.68	1484	13.37
12	Cardiac and Cardiovascular Systems	94	3.11	2640	28.09
13	Health Care Sciences and Services	82	2.72	1308	15.95
14	Oncology	81	2.68	1450	17.90
15	Gerontology	69	2.28	795	11.52
16	Clinical Neurology	51	1.69	604	11.84
17	Respiratory System	46	1.52	408	8.87
18	Medicine, Research and Experimental	45	1.49	399	8.87
19	Psychology	45	1.49	642	14.27
20	Psychology, Applied	41	1.36	511	12.46

TP, total papers; TRP%, percent of total articles in the field; TC, total citations; ACPP, average citations per publication.

**Table 4:** Top 20 journals publishing papers in sedentary time research.

Rank	Journal Title	TP	TC	ACPP	IF
1	*BMC Public Health*	170	3438	20.22	2.521
2	*Int. J. Behav. Nutr. Phys. Act.*	163	7152	43.88	6.714
3	*PLOS One*	156	4338	27.81	2.740
4	*Int. J. Environ. Res. Public Health*	136	852	6.26	2.849
5	*Med. Sci. Sports Exerc.*	128	4452	34.78	4.029
6	*J. Phys. Act. Health*	122	1425	11.68	1.993
7	*Prev. Med.*	61	2492	40.85	3.788
8	*BMJ Open*	56	702	12.76	2.496
9	*J. Sports Sci.*	52	371	7.13	2.597
10	*Scand. J. Med. Sci. Sports*	46	432	9.39	3.255
11	*J. Sci. Med. Sport*	45	934	20.76	3.607
12	*Pediatr. Exerc. Sci.*	38	400	10.53	1.489
13	*Am. J. Prev. Med.*	37	3225	87.16	4.420
14	*Int. J. Obes.*	32	811	25.34	4.419
15	*Obesity*	29	734	25.31	3.742
16	*J. Aging Phys. Act.*	26	222	8.54	1.763
17	*Appl. Physiol. Nutr. Metab.*	25	423	16.92	2.522
18	*Br. J. Sports Med.*	23	1459	63.43	12.68
19	*J. Occup. Environ. Med.*	20	267	13.35	1.642
20	*Nutrients*	20	91	4.55	4.546

TP, total papers; IF, Impact Factor 2019; TC, total citations; ACPP, average citations per publication.

**Table 5:** Contribution of the top 20 authors in sedentary time research.

Rank	Author	TP	TAR	TC	ACPP	H-Index	Institution (Current), Country/Region
1	Ekelund U.	99	12	4063	41.04	31	Norwegian Sch Sport Sci, Norway
2	Owen N.	88	3	7698	87.48	43	Baker Heart and Diabetes Institute, Australia
3	Dunstan D.W.	84	4	6782	80.74	38	Baker Heart and Diabetes Institute, Australia
4	Healy G.N.	64	9	6244	97.56	36	Univ Queensland, Australia
5	Brage S.	61	5	1603	26.28	23	Univ Cambridge, UK
6	Tremblay M.S.	60	6	4141	69.02	28	Children’s Hosp Eastern Ontario, Canada
7	De Bourdeaudhuij I.	55	3	1711	31.11	23	Univ Ghent, Belgium
8	Yates T.	55	4	2666	49.37	20	Univ Leicester, UK
9	Salmon J.	53	7	1724	32.53	23	Deakin Univ, Australia
10	Chaput J.P.	48	11	1758	36.63	23	Childrens Hosp Eastern Ontario, Canada
11	Sardinha L.B.	46	15	1236	26.87	21	Univ Lisbon, Portugal
12	Katzmarzyk P.T.	44	4	1428	32.45	18	Pennington Biomed Res Ctr, USA
13	Edwardson C.L.	43	11	2332	55.52	18	Univ Leicester, UK
14	Olds T.	43	1	1259	29.28	20	Univ S Australia, Australia
15	Cardon G.	42	20	1124	26.76	19	Univ Ghent, Belgium
16	Davies M.J.	42	2	2085	50.85	17	Univ Leicester, UK
17	Khunti K.	38	0	2071	54.50	18	Univ Leicester, UK
18	Andersen L.B.	36	2	697	19.36	17	Western Norway Univ Appl Sci, Norway
19	Hamer M.	35	15	952	27.20	19	UCL, UK
20	Kerr J.	35	4	604	17.26	15	Univ Calif San Diego, USA

TP, total papers; TAR, total number of articles for which they are responsible; TC, total citations; ACPP, average citations per publication.

**Table 6:** Yearly most cited publications during the period of 2010–2020.

Year	Authors	Title	TC	TCY	Source	Country/Region
2010	Owen N. et al.	Too Much Sitting: The Population Health Science of Sedentary Behavior	1214	110	*Exerc. Sport Sci. Rev.*	Australia; USA
2011	Tremblay M.S. et al.	Systematic review of sedentary behaviour and health indicators in school-aged children and youth	969	97	*Int. J. Behav. Nutr. Phys. Act.*	Canada; USA
2012	Wilmot E.G. et al.	Sedentary time in adults and the association with diabetes, cardiovascular disease and death: systematic review and meta-analysis	859	95	*Diabetologia*	England
2013	Peddie M.C. et al.	Breaking prolonged sitting reduces postprandial glycemia in healthy, normal-weight adults: a randomized crossover trial	227	28	*Am. J. Clin. Nutr.*	New Zealand
2014	Dyrstad S.M. et al.	Comparison of Self-reported versus Accelerometer-Measured Physical Activity	305	44	*Med. Sci. Sports Exerc.*	Norway
2015	Biswas A. et al.	Sedentary Time and Its Association With Risk for Disease Incidence, Mortality, and Hospitalization in Adults A Systematic Review and Meta-analysis	1141	190	*Ann. Intern. Med.*	Canada
2016	Warburton D.E.R. et al.	Reflections on Physical Activity and Health: What Should We Recommend?	171	34	*Can. J. Cardiol.*	Canada
2017	Tremblay M.S. et al.	Sedentary Behavior Research Network (SBRN)—Terminology Consensus Project process and outcome	811	203	*Int. J. Behav. Nutr. Phys. Act.*	Canada; Scotland; Belgium; Netherlands
2018	Patterson R. et al.	Sedentary behaviour and risk of all-cause, cardiovascular and cancer mortality, and incident type 2 diabetes: a systematic review and dose response meta-analysis	220	73	*Eur. J. Epidemiol.*	England; Brazil
2019	Ekelund U. et al.	Dose–response associations between accelerometry measured physical activity and sedentary time and all cause mortality: systematic review and harmonised meta-analysis	167	84	*BMJ-British Medical Journal*	Norway; USA; Sweden
2020	Huckins J.F. et al.	Mental Health and Behavior of College Students During the Early Phases of the COVID-19 Pandemic: Longitudinal Smartphone and Ecological Momentary Assessment Study	38	38	*J. Med. Internet Res.*	USA

TC, total citations; TCY, total citations per year.

## Data Availability

All data generated or analyzed during this study are included in this published article.
